# The Interaction between Early Life Epilepsy and Autistic-Like Behavioral Consequences: A Role for the Mammalian Target of Rapamycin (mTOR) Pathway

**DOI:** 10.1371/journal.pone.0035885

**Published:** 2012-05-02

**Authors:** Delia M. Talos, Hongyu Sun, Xiangping Zhou, Erin C. Fitzgerald, Michele C. Jackson, Peter M. Klein, Victor J. Lan, Annelise Joseph, Frances E. Jensen

**Affiliations:** 1 Department of Neurology, Children’s Hospital, Boston, Massachusetts, United States of America; 2 Division of Neuroscience, Children’s Hospital, Boston, Massachusetts, United States of America; 3 Program in Neuroscience, Harvard Medical School, Boston, Massachusetts, United States of America; University of Utah, United States of America

## Abstract

Early life seizures can result in chronic epilepsy, cognitive deficits and behavioral changes such as autism, and conversely epilepsy is common in autistic children. We hypothesized that during early brain development, seizures could alter regulators of synaptic development and underlie the interaction between epilepsy and autism. The mammalian Target of Rapamycin (mTOR) modulates protein translation and is dysregulated in Tuberous Sclerosis Complex, a disorder characterized by epilepsy and autism. We used a rodent model of acute hypoxia-induced neonatal seizures that results in long term increases in neuronal excitability, seizure susceptibility, and spontaneous seizures, to determine how seizures alter mTOR Complex 1 (mTORC1) signaling. We hypothesized that seizures occurring at a developmental stage coinciding with a critical period of synaptogenesis will activate mTORC1, contributing to epileptic networks and autistic-like behavior in later life. Here we show that in the rat, baseline mTORC1 activation peaks during the first three postnatal weeks, and induction of seizures at postnatal day 10 results in further transient activation of its downstream targets phospho-4E-BP1 (Thr37/46), phospho-p70S6K (Thr389) and phospho-S6 (Ser235/236), as well as rapid induction of activity-dependent upstream signaling molecules, including BDNF, phospho-Akt (Thr308) and phospho-ERK (Thr202/Tyr204). Furthermore, treatment with the mTORC1 inhibitor rapamycin immediately before and after seizures reversed early increases in glutamatergic neurotransmission and seizure susceptibility and attenuated later life epilepsy and autistic-like behavior. Together, these findings suggest that in the developing brain the mTORC1 signaling pathway is involved in epileptogenesis and altered social behavior, and that it may be a target for development of novel therapies that eliminate the progressive effects of neonatal seizures.

## Introduction

Epilepsy is the third most common major neurological disease [1], [2] and is increasingly recognized as a disease that reaches well beyond seizures, with a high incidence of neuropsychiatric co-morbidities not associated with abnormal electrographic activity. Up to half of all epilepsy patients suffer cognitive and/or neuropsychiatric disabilities [3], [4]. Nowhere is this more prominent than in early life, where the incidence of seizures is at one of highest levels of the lifespan [5]. In addition, early life epilepsy is often accompanied by learning and neurocognitive disorders, including autism [6], [7]. In the case of autism, epilepsy is present in up to 46% of patients, and correlates with lower IQ [8]. Co-occurrence of epilepsy and autism has been found in approximately 30% of children with either disorder [8], [9], [10] suggesting an interaction between epilepsy and autism.

Rodent models of early life seizures also exhibit long term consequences of epilepsy and altered synaptic plasticity [11]. Clinically, the most common cause of seizures in the neonatal period is hypoxic/ischemic encephalopathy [12]. The rat model of neonatal hypoxia-induced seizures (HS) demonstrates features of the human disease state, including post-seizure changes in hippocampal and cortical excitability [11], [13], increased later life seizure susceptibility, cognitive deficits, mossy fiber sprouting, and spontaneous seizures [14], [15]. In addition, neonatal seizures cause early post-translational modification and potentiation of the α-Amino-3-hydroxy-5-Methyl-4-isoxazole-Propionic Acid (AMPA) subtype of excitatory glutamate receptors similar to those observed in physiologic synaptic plasticity [11], [16].

We hypothesize that during a period of robust synaptogenesis, seizures may cause age-specific alterations that result in both epilepsy and neurobehavioral deficits. As synaptic plasticity models demonstrate that long-lasting change requires new protein synthesis [17], we investigated whether mTOR-dependent protein translation was altered following neonatal seizures, thereby contributing to epileptogenesis and behavioral deficits.

The mTOR Complex 1 (mTORC1) pathway regulates protein translation via activation of the ribosomal S6 protein kinase (p70S6K) and its downstream target ribosomal S6 protein, as well as inactivation of the eukaryotic initiation factor 4E (eIF4E)-binding protein 1 (4E-BP1). The mTORC1 is activated by many factors, including ionotropic and metabotropic glutamate receptors, trophic factors such as brain-derived neurotrophic factor (BDNF), activity dependent extracellular signal-regulated kinase (ERK) pathway, and phosphoinositide-3′ kinase (PI3K)/Akt pathway, and is constitutively suppressed by the TSC1 (hamartin) and TSC2 (tuberin) complex [17], [18]. Inactivating mutations in either *TSC1* or *TSC2* result in Tuberous Sclerosis Complex (TSC), characterized by abnormal cortical development and seizures [19]. Epilepsy occurs in up to 80% of TSC cases and intellectual disability and/or autism is seen in up to 60% [7], [20], [21], [22]. Mutations of *Tsc1* or *Tsc2* in the mouse are associated with seizures, cognitive disorders and autistic-like behavior [23], [24], [25], [26], [27].

The mTORC1 pathway critically regulates neuronal growth, synaptic plasticity and memory consolidation [28], [29], and inhibition of mTORC1 with rapamycin blocks long term synaptic potentiation (LTP), a model of learning and memory [30]. In adult brain, recent studies have shown that the mTORC1 pathway can be activated by seizures in the absence of a genetic mutation in *Tsc1/Tsc2* [31], [32], [33], [34].

Despite the clinical link between epilepsy and neurobehavioral deficits in TSC and the experimental evidence that mTORC1 plays a role in epileptogenesis in adulthood, little is known about whether seizure-induced changes in mTORC1 signaling occur in early life epilepsy, or whether these changes have behavioral and epileptogenic consequences [35]. To investigate the role of mTORC1 in later epileptogenesis and behavioral deficits without the confound of cell death seen in adult models, we used a model of early life hypoxic seizures [15], [36]. We first assessed baseline and seizure-induced changes of critical downstream and upstream components of mTORC1 signaling in the developing brain. Next, we examined whether these changes were associated with altered glutamate receptor function, later life epilepsy and social behavioral deficits, given the high incidence of these abnormalities in genetic diseases that upregulate mTORC1 pathway activity. Finally, we determined whether early treatment with the mTORC1 inhibitor rapamycin could attenuate subsequent behavioral deficits and seizure-induced increases in neuronal excitability *in vivo* and *in vitro.*

## Results

### The mTORC1 Pathway Activity is Upregulated in the Developing Brain

**Figure 1:**
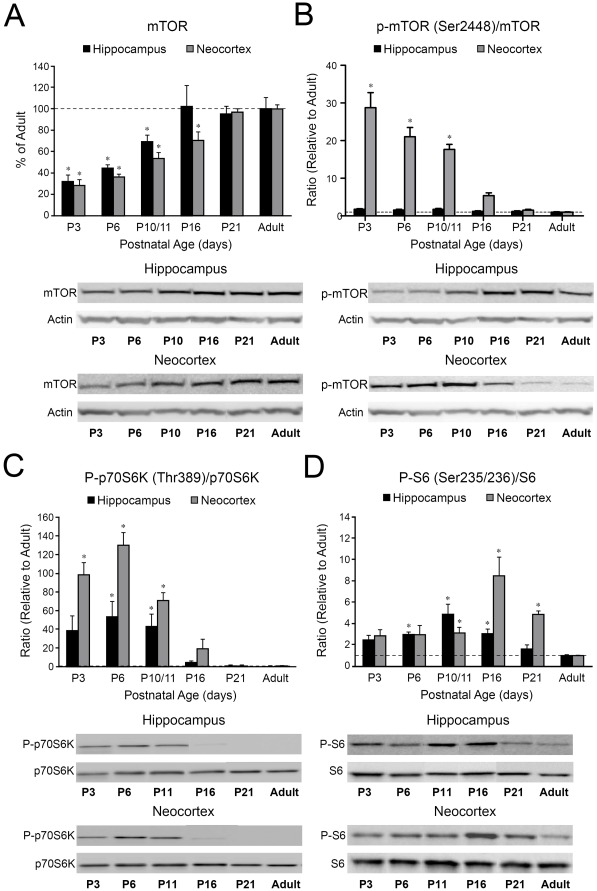
Components of the mTORC1 pathway are developmentally regulated. A. Western blot quantification of total mTOR protein levels in rat brain demonstrates a significant downregulation at P3–P11 in the hippocampus (32.0±6.1% of adult, n = 5, p<0.01 at P3, 44.7±2.8 of adult, n = 4, p<0.05 at P6, 69.0±6.1% of adult, n = 5, p<0.05 at P10/11) and P3–P16 in the neocortex (28.0±5.4% of adult, n = 4, p<0.001 at P3, 36.4±2.1 of adult, n = 4, p<0.001, 54.0±5.2% of adult, n = 5, p<0.001 at P10/11, 70.0±7.8% of adult, n = 4, p<0.05 at P16), compared to adulthood (100%). B. Phospho-mTOR (Ser2448)/mTOR ratios demonstrate a modest peak at P3 in the hippocampus (1.76±0.14 fold, n = 5, p>0.05), and a significant upregulation at P3–P10/11 in the neocortex (28.8±4.5 fold increase, n = 4, p<0.001 at P3, 21.0±2.5 fold increase, n = 4, p<0.001 at P6, 17.7±1.7 fold increase, n = 5, p<0.001 at P10/11), as compared to adult standard (1). C. Phospho-p70S6K (Thr389)/p70S6K ratios demonstrate a significant upregulation at P6–P11 in the hippocampus (54.2±15.3 fold increase, n = 4, p<0.05 at P6, 43.8±12.3 fold increase, n = 5, p<0.05 at P10/11) and at P3–P11 in the neocortex (98.5±12.6 fold, n = 5, p<0.001 at P3, 130.3±12.6 fold, n = 4, p<0.001 at P6, 70.7±8.4 fold, n = 7, p<0.001 at P10/11) as compared to adult standard (1). D. Phospho-S6 (Ser235/236)/S6 ratios are significantly increased at P6–P16 in the hippocampus (2.9±0.2 fold increase, n = 5, p<0.001 at P6, 4.9±0.9 fold increase, n = 5, p<0.001 at P10/11, 3.0±0.3 fold increase, n = 4, p<0.01 at P16), and at P10–P21 in the neocortex (3.1±0.4 fold, n = 5, p<0.05 at P10/11, 8.5±1.7 fold, n = 5, p<0.01 at P16, 4.9±0.3 fold, n = 4, p<0.001 at P21), relative to adult standard (1). Mean values for each age group are expressed relative to the mean adult levels, represented by the dotted line. *: p<0.05. Error bars indicate S.E.M. Insets are the representative western blots for individual proteins.

As the mTORC1 signaling is known to control neuronal maturation and plasticity, we hypothesized that mTORC1 activity is enhanced during early postnatal development. First, we quantified the expression of total mTOR protein and found that it gradually increased from postnatal day 3 (P3) to P10/11 in both hippocampus and neocortex (p<0.05), before approaching almost adult levels by P16–P21 (Figure 1A). Phospho-mTOR (Ser2448), which can be associated with both mTORC1 and mTORC2 [37] (Figure 1B), demonstrated different trends in hippocampus and neocortex: it progressively increased with age in the hippocampus (p>0.05), while being transiently elevated between P3–P10/11 before dropping to adult levels in the neocortex (p<0.001). Phospho-mTOR (Ser2448)/mTOR ratios demonstrated no maturational changes in the hippocampus, but a significant upregulation at P3–P10/11 in the neocortex (p<0.001), as compared to adult standard. Due to the fact that phosphorylation of Ser2448 has been linked to both mTORC1 and mTORC2, in order to assess the mTORC1 pathway specifically, we quantified the phosphorylation status of two of its major downstream targets: phospho-p70S6K (Thr389) and phospho-S6 (Ser235/236) [17], [38]. Unlike endogenous mTOR protein, total p70S6K and S6 showed stable expression levels over time, however both were highly phosphorylated at early ages (Figure 1C–D). Phospho-p70S6K/p70S6K ratios showed a transient upregulation with significantly increased levels at P6–P10/11 in hippocampus (p<0.05), and at P3–P10/11 in neocortex (p<0.001) (Figure 1C). Similarly, phospho-S6/S6 ratios in the hippocampus were upregulated at P6 (p<0.001), P10/11 (p<0.001) and at P16 (p<0.01), and relatively delayed in neocortex, with highest levels occurring at P10/11 (p<0.05), P16 (p<0.01) and P21 (p<0.001) (Figure 1D). These results demonstrate that, although total mTOR gradually increases with maturation, functional markers of mTORC1 activity are transiently elevated during early brain development coincident with the peak of synaptogenesis [16], [39].

**Figure 2:**
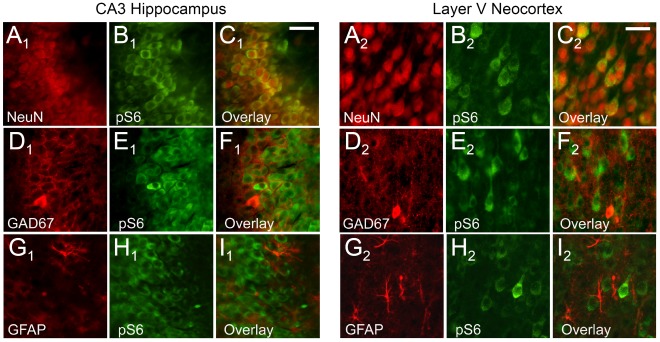
Cell-specific expression of the phospho-S6 (Ser235/236) protein in the normal developing rat brain. Coronal sections double labeled with NeuN (red) and phospho-S6 (Ser235/236) (green) demonstrate high phospho-S6 expression in hippocampal CA3 region (A1–C1) and neocortical layer V (A2–C2) at P11. Double labeling with GAD-67 (red) in combination with phospho-S6 (green) demonstrates no co-expression in hippocampal (D1–F1) or neocortical (D2–F2) interneurons. GFAP and phospho-S6 double labeling shows no phospho-S6 expression in the hippocampal (G1–I1) and neocortical (G2–I2) astrocytes. Scale bars 50 µm.

Double-labeling immunocytochemistry with the neuronal marker NeuN and phospho-S6 (Ser235/236) confirmed neuronal co-localization, both in hippocampus (Figure 2A1–C1) and neocortex (Figure 2A2–C2) (n = 6). In contrast, there was no co-localization of phospho-S6 with either the GABA-ergic neuronal marker GAD-67 (Figure 2D1–F1, D2–F2), or the astrocytic marker GFAP (Figure 2G1–I1, G2–I2). This demonstrates that the developmental hyperactivation of mTORC1 pathway seen by western blots predominates in excitatory principal neurons, with little contribution of inhibitory neurons or astroglia.

### mTORC1 Signaling Pathway is Further Transiently Activated by Neonatal Seizures

**Figure 3:**
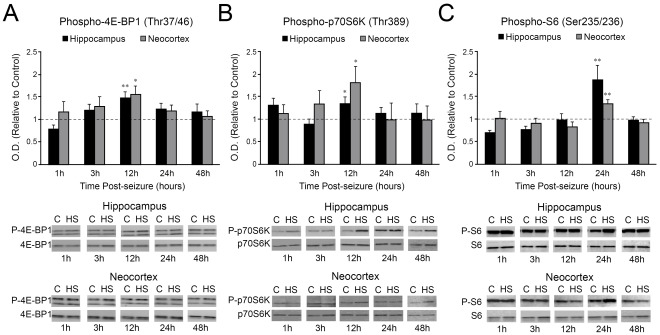
Neonatal seizures transiently activate the mTORC1 downstream targets phospho-4E-BP1 (Thr37/46), phospho-p70S6K (Thr389), and phospho-S6 (Ser235/236). A. Phospho-4E-BP1 levels significantly increase in both hippocampus (1.47±0.13 fold, n = 12, p<0.001) and neocortex (1.55±0.19 fold, n = 11, p<0.05) at 12 h after HS induction in P10 rat pups. B. Phospho-p70S6K is simultaneously significantly upregulated in both hippocampus (1.34±0.14 fold, n = 7, p<0.05) and neocortex (1.81±0.34 fold, n = 7, p<0.05) 12 h following HS onset. C. Subsequent increases in phospho-S6 are observed in both hippocampus (1.88±0.31 fold, n = 12, p<0.01) and neocortex (1.35±0.08 fold increase, n = 9, p<0.01) at 24 h post-HS. Histograms represent averaged optical density normalized to actin, and expressed relative to the mean control values (represented by the dotted line). No significant difference is observed in the total levels of any of these proteins (p>0.05). Insets are the representative western blots for individual proteins. C: control; HS: hypoxic seizures. *: p<0.05, **: p<0.01. Error bars indicate S.E.M.

Changes in the mTORC1 downstream targets phospho-4E-BP1 (Thr37/46), phospho-p70S6K (Thr389) and phospho-S6 (Ser235/236) were quantified over the first 48 h after hypoxic seizures (HS) induced at P10 (Figure 3). At 12 h post-HS, phospho-4E-BP1 increased in both hippocampus (p<0.001) and neocortex (p<0.05, Figure 3A), with similar increases in phospho-p70S6K (p<0.05, Figure 3B). Phospho-S6 upregulation followed later at 24 h after HS in both hippocampus and neocortex (p<0.01, Figure 3C). There were no significant changes in total levels of 4E-BP1, p70S6K and S6 (p>0.05, Figure 3). These results demonstrate that seizures in the developing brain cause acute increases in downstream components of mTORC1 pathway.

### Neonatal Seizures Induce Transient Upregulation of BDNF Levels and Early Activation of PI3- and MAP- Kinase Signaling Pathways

**Figure 4:**
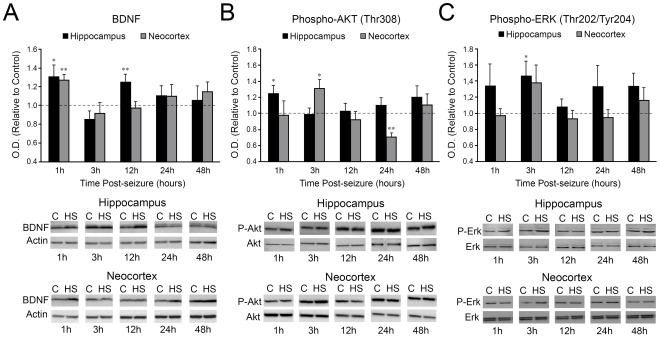
Rapid seizure-induced alterations in mTORC1 upstream regulators BDNF, phospho-Akt (Thr308) and phospho-ERK1/2 (Thr202/Tyr204). A. Quantification of BDNF protein levels at 1, 3, 12, 24, 48 h post-HS shows a bi-phasic upregulation in the hippocampus at 1 h (1.31±0.12 fold, n = 7, p<0.05) and 12 h (1.25±0.08 fold, n = 7, p<0.01), and a single increase at 1 h (1.27±0.06 fold, n = 10, p<0.01) in the neocortex. Of note, only the mature 14 kD BDNF band is quantified because of the relevance of this isoform for synaptic plasticity and epileptogenesis. B. Phospho-Akt (Thr308) is significantly increased at 1 h post-HS in the hippocampus (1.24±0.09 fold, n = 8, p<0.05), while in the cortex levels are upregulated at 3 h (1.3±0.11 fold, n = 7, p<0.05) and downregulated at 24 h after HS (0.7±0.04 fold, n = 7, p<0.01). C. Phospho-ERK1/2 expression is significantly higher at 3 h post-HS in the hippocampus (1.46±0.18 fold, n = 7), but not in the neocortex (1.37±0.22 fold, n = 11, p = 0.11). Averaged optical density normalized to actin are expressed relative to the mean control values (dotted line). No significant difference is observed in the total levels of phospho-Akt (Thr308) or phospho-ERK1/2 (Thr202/Tyr204) (p>0.05). Insets are the representative western blots for individual proteins. C: control; HS: hypoxic seizures. *: p<0.05, **: p<0.01. Error bars indicate S.E.M.

Brain derived neurotrophic factor (BDNF), which can trigger mTORC1-dependent protein translation in activity-dependent neuronal plasticity [29], [40], was transiently increased at 1 h (p<0.05) and 12 h (p<0.01) following seizures in hippocampus, and at 1 h in neocortex (p<0.005, Figure 4A). The kinases Akt and ERK1/2, downstream from BDNF, and upstream from mTORC1 [41] showed a rapid induction following neonatal seizures. Phospho-Akt (Thr308) levels were transiently elevated at 1 h after HS in hippocampus (p<0.05) and 3 h in neocortex (p<0.05), with later neocortical downregulation at 24 h post-HS (p<0.01, Figure 4B). Phospho-ERK1/2 (Thr202/Tyr204) levels were briefly increased at 3 h post-HS, more consistent in hippocampus (p<0.05), compared to neocortex showing variable responses (p = 0.11) (Figure 4C). Importantly, total Akt and ERK1/2 levels remained unaltered in hippocampus and in neocortex compared to controls during this time course (p>0.05, Figure 4B-C). These data indicate that HS in the immature brain induce a rapid and transient activation of BDNF, followed by PI3K/Akt/mTORC1 and the ERK1/2 signaling pathways.

### Cell-specific Upregulation of mTORC1 Signaling Following Neonatal Seizures

**Figure 5:**
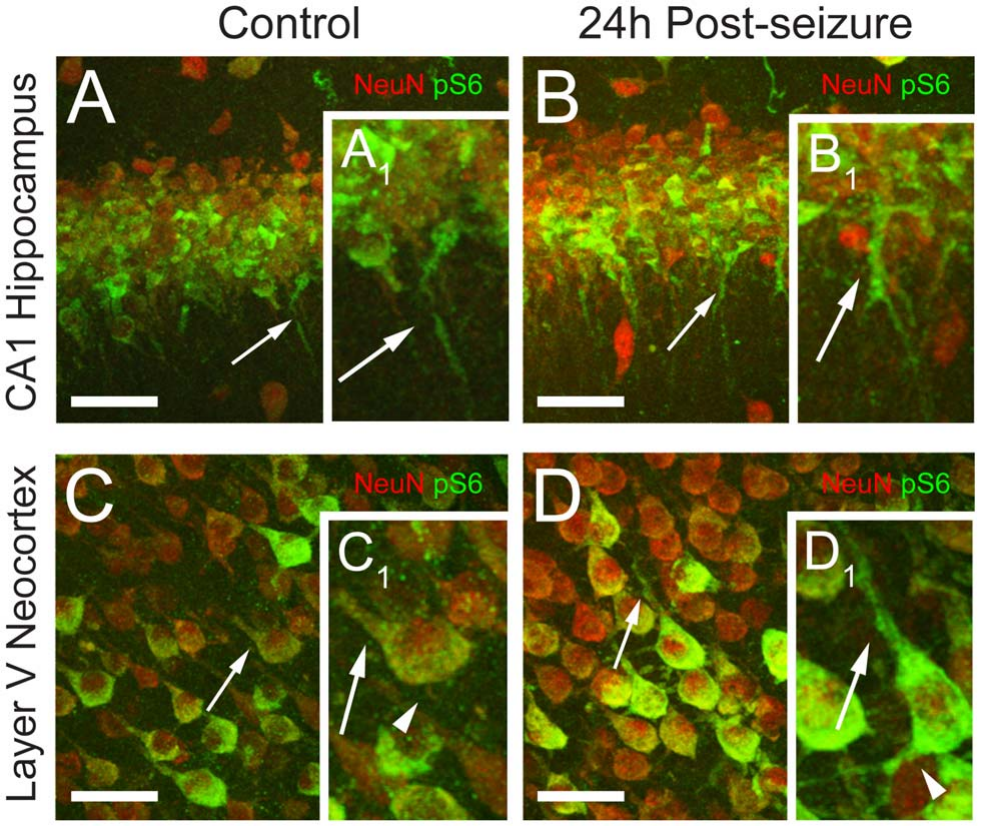
Phospho-S6 (Ser235/236) expression is highly upregulated in the cell bodies and dendrites of hippocampal and neocortical pyramidal neurons at 24 h following neonatal seizures. A-B. Confocal images of CA1 hippocampal region double labeled with NeuN (red) and phospho-S6 (green) show a moderate increase in phospho-S6 expression in pyramidal neuron cell bodies and a marked phospho-S6 upregulation in the dendrites (arrows) in the post-HS group (B, B1), relative to controls (A, A1). C-D. Confocal microscopy of neocortical layer V demonstrates a similar predominant increase in phospho-S6 (green) expression in the apical (arrows) and basal (arrowheads) dendrites of NeuN (red) positive neurons in the HS group (D, D1), relative to controls (C, C1). Confocal images represent z-stacks composed of multiple plane images collected at 1 µm intervals. Scale bars are 100 µm for A-D. Insets (A1–D1) show corresponding higher magnification of individual cells.

We next examined the cellular localization of the mTORC1 pathway marker phospho-S6 at the 24 h peak of the post-seizure activation (n = 6/group). We observed an overall increase in phospho-S6 expression in pyramidal neurons, most prominent in the apical and basal dendrites (Figure 5). SMI 311 neurofilament staining was unchanged post-HS, suggesting no changes in dendrite morphology or integrity. In addition, no seizure-induced increases in phospho-S6 were observed in either interneurons or astrocytes (data not shown), suggesting that mTORC1 activation is predominantly induced in the dendritic portion of glutamatergic neurons, consistent with its known role in synaptic function [17].

### Rapamycin Treatment Attenuates Seizure-induced Increases in 4E-BP1, p70S6K and S6 Phosphorylation

**Figure 6:**
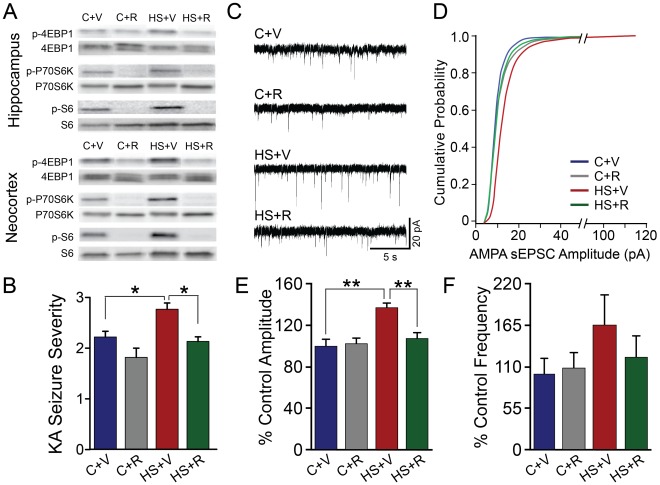
Rapamycin attenuates seizure-induced mTORC1 pathway activation, subsequent subacute increases in seizure susceptibility, and enhanced AMPA receptor function. A. Representative western blots of hippocampal and neocortical tissue, collected at 12–24 h post-HS, show that rapamycin treatment almost completely blocks phospho-4EBP1, phospho-p70S6K and phospho-S6 in HS and normoxic control animals, with no effect on total protein levels. The rapamycin-treated HS rats (HS+R) demonstrate a significant reduction of phospho-4EBP1/4EBP1 (0.48±0.07, n = 8, p<0.05 in hippocampus, and 0.52±0.04, n = 8, p<0.01 in neocortex), phospho-p70S6K/p70S6K (0.43±0.04, n = 4, p<0.05 in hippocampus, and 0.19±0.06, n = 8, p<0.001 in neocortex), and phospho-S6/S6 (0.03±0.02, n = 4, p<0.001 in hippocampus, and 0.08±0.02, n = 4, p<0.001 in neocortex), relative to vehicle-treated controls (C+V) (1). Sham control rats treated with rapamycin (C+R) also show a significant decrease in phospho-4EBP1/4EBP1 (0.56±0.06, n = 8, p<0.05 in hippocampus, and 0.53±0.04, n = 8, p<0.01 in neocortex), phospho-p70S6K/p70S6K (0.45±0.05, n = 4, p<0.05 in hippocampus, and 0.16±0.01, n = 8, p<0.001 in neocortex), and phospho-S6/S6 (0.1±0.02, n = 4, p<0.001 in hippocampus, and 0.08±0.03, n = 4, p<0.001 in neocortex), compared to vehicle-treated sham controls (C+V). B. Rapamycin treatment reduces subacute increases in seizure susceptibility induced by kainic acid (KA) administration. When exposed to KA at P13, vehicle-treated HS rats (HS+V) demonstrate a significant increase in the average seizure severity (2.8±0.12, n = 13; modified Racine scale, see methods), compared to vehicle-treated normoxic control littermates (C+V), (2.2±0.11, n = 27, p<0.05). Rapamycin-treated HS rats (HS+R) show a significant decrease in KA seizure severity, relative to the HS+V group (2.13±0.09, n = 15; p<0.01). C. Representative traces of AMPA receptor-mediated spontaneous excitatory post-synaptic currents (sEPSCs), pharmacologically isolated by blocking NMDA and GABA_A_ receptors, in hippocampal *ex vivo* slices from control and HS rats treated *in vivo* with either vehicle (C+V, HS+V) or rapamycin (C+R, HS+R) and removed at 48 h post-HS (P12). D. Normalized cumulative distribution of AMPA receptor sEPSCs, recorded at 48 h post-HS demonstrates a significant increase in amplitude in the vehicle-treated HS (HS+V) animals compared to vehicle (C+V) or rapamycin-treated controls (C+R), and this is significantly attenuated by *in vivo* rapamycin treatment (HS+R), (p<0.001, Kolmogorov–Smirnov test). E. AMPA receptor sEPSCs amplitude is significantly higher in the vehicle-treated HS group (HS+V), relative to vehicle-treated controls (C+V), (137.62±3.39% of vehicle-treated controls, n = 6; p<0.001), and reduced significantly in the rapamycin-treated HS rat pups (HS+R), (107.93±4.64% of vehicle-treated controls, n = 6; p<0.01). *In vivo* rapamycin treatment has no effect on AMPA receptor sEPSCs amplitude in the normoxic control rats (C+R), (102.28±5.4% of vehicle-treated controls; n = 6, p>0.05). F. AMPA receptor sEPSCs show a trend of increased frequency in the HS+V group, compared to C+V and C+R groups (164.98±37.98% of vehicle-treated controls, n = 6, p = 0.16), which is attenuated by *in vivo* rapamycin treatment (122.35±26.5% of vehicle-treated controls, n = 6). *In vivo* rapamycin treatment has no effect on AMPA receptor sEPSCs frequency in the normoxic control rats (C+R) (108.39±18.6% of vehicle-treated controls, n = 6, p>0.05). *: p<0.05; **: p<0.01. Error bars indicate S.E.M.

Rapamycin (3 mg/kg i.p.) was administered 24 h before and 1h post-HS in order to achieve trough levels of phospho-S6 at the time of exposure to hypoxia at P10 (see Methods). This treatment markedly suppressed the post-HS increases in phospho-4E-BP1, phospho-p70S6K and phospho-S6 in both hippocampus and neocortex (Figure 6A). Specifically, rapamycin-treated HS rats demonstrated a significant reduction of phospho-4EBP1/4EBP1 (p<0.05 in hippocampus, and p<0.01 in neocortex), phospho-p70S6K/p70S6K (p<0.05 in hippocampus, and p<0.001 in neocortex), and phospho-S6/S6 ratios (p<0.001 in hippocampus, and p<0.001 in neocortex), relative to vehicle-treated controls. In addition, consistent with prior reports in normal brain [42], [43], this dose also suppressed baseline activity of mTORC1 downstream markers in naïve control rat pups (Figure 6A), confirming pharmacologic efficacy and brain penetration of this agent. Rapamycin-treated control rats demonstrated a significant decrease in phospho-4EBP1/4EBP1 (p<0.05 in hippocampus, and p<0.01 in neocortex), phospho-p70S6K/p70S6K (p<0.05 in hippocampus, and p<0.001 in neocortex), and phospho-S6/S6 ratios (p<0.001 in hippocampus and neocortex), compared to vehicle- treated sham controls.

### Rapamycin Treatment Protects against Subacute Increases in Seizure Susceptibility and Enhanced AMPA Receptor Function

Neonatal seizures are characterized by subacute increases in susceptibility to kainic acid (KA) seizures [11], [36], and by subsequent development of chronic epilepsy [15]. As there is no treatment to date to prevent the long term epileptogenesis after early life seizures [10], [44], we examined whether mTORC1 inhibition may have a protective effect. Control and HS rats were treated with rapamycin or vehicle as previously described, and then seizures were induced by systemic KA injection at P13 (72 h post-HS, 2 mg/kg, i.p.). Consistent with previous reports [32], [33], rapamycin had no acute effects on the initial HS at P10, as it did not alter either the number of generalized tonic-clonic seizures per rat pup (vehicle: 9.84±1.07, n = 13; rapamycin: 8.13±1.16, n = 15; p = 0.29), or the average duration of these seizures (vehicle: 17.06±3.59 sec, n = 12; rapamycin: 15.37±2.37 sec, n = 12; p = 0.70). However, rapamycin treatment reversed the enhanced susceptibility to KA seen in HS rats at P13 (Figure 6B). As we have previously shown [11], [36], KA-induced seizure severity (maximal seizure stage reached) at P13 was increased in vehicle-treated HS rats relative to vehicle-treated littermate controls (p<0.05) (Figure 6B). In contrast, rapamycin-treated HS animals exhibited a significantly lower seizure severity score, compared to the vehicle-treated HS rats (p<0.05), and this was similar to the vehicle-treated naïve control group (Figure 6B). The KA seizure severity was unchanged in the rapamycin-treated control group (p>0.05). Additionally, we found no relationship between the number of HS and the severity of KA-induced seizures in either the vehicle- treated or rapamycin-treated HS rats (p = 0.72; two-way ANOVA).

Concurrent with increased seizure susceptibility at P13 *in vivo*, we have also reported a subacute increase in AMPA receptor function in hippocampal CA1 neurons in *ex vivo* slices removed at 48–72 h post-HS [16]. As mTORC1 activation upregulates AMPA receptor function *in vitro* [28], we tested whether HS-induced enhancement of receptor function could be prevented by *in vivo* rapamycin treatment. Spontaneous (s) AMPA receptor-mediated EPSCs (pharmacologically isolated by blocking GABA and NMDA receptors with 60 µM picrotoxin and100 µM DL-AP-5, respectively) were recorded in hippocampal CA1 pyramidal neurons in slices removed 48 h post-HS. Neurons from vehicle-treated HS group showed significantly larger sEPSCs amplitude compared to vehicle-treated littermate controls (p<0.001), as well as a trend toward higher frequencies (p = 0.16) (Figure 6C–F), consistent with our prior results [16]. *In vivo* treatment with rapamycin reversed seizure-induced enhancement of AMPA receptor-mediated sEPSC amplitude (p<0.01) in slices removed from HS rats, but had no significant effect in the naïve littermate controls (p>0.05, Figure 6C–F). AMPA receptor-mediated miniature (m) EPSCs, isolated by adding TTX (1 µM) to the bath solution, demonstrated a similar change at 48 h post-HS. The amplitude of AMPA mEPSCs was significantly increased in vehicle-treated HS animals compared to the vehicle-treated naïve controls (139.9±8.1% of vehicle-treated controls, p<0.01, n = 5). Rapamycin treatment significantly attenuated the increased mEPSC amplitude in HS rats (103.5±5.4% of vehicle-treated controls, p<0.01, n = 5), but showed no effects in the naïve controls (101.2±4.1% of vehicle-treated controls, p>0.05, n = 5). These results suggest a potential role of mTORC1 activation in the increased seizure susceptibility and enhancement of AMPA receptor function observed following early life seizures.

### mTORC1 Inhibition Prevents the Development of Chronic Epilepsy Following Neonatal Seizures

**Figure 7 pone-0035885-g007:**
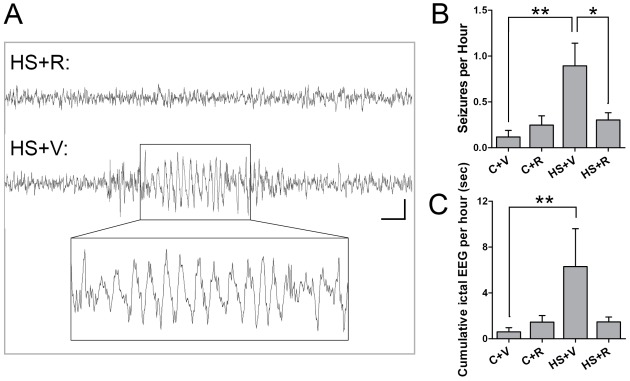
Rapamycin treatment at HS attenuates later epilepsy. A. Representative EEG traces from HS+R and HS+V rats, with HS+V showing an example of an observed seizure. Scale bar  = 1 sec, 50 µV for both traces. Inset shows an expanded view of the EEG trace in the HS+V group. B. Subdermal wire electrode EEG recordings show that rats that had received vehicle with HS at P10 (HS+V) have significantly more spontaneous seizures at P36–38 (0.89±0.25 seizures/h, n = 15) than control normoxic litter mates (C+V), (0.12±0.07 seizures/h, n = 16, p<0.01). Rapamycin treatment at HS (HS+R) attenuates the HS induced later life epilepsy (0.25±0.10 seizures/h, n = 15, p<0.05). Rapamycin treatment of sham P10–11 littermates (C+R) has no significant effect on the EEG pattern (0.30±0.08 seizures/h, n = 28, p = 0.70), as compared to the C+V. C. Rapamycin also reverses the effects of HS on cumulative ictal EEG activity/h. Rats with prior HS treated with vehicle (HS+V) exhibit significantly greater ictal activity per hour (6.30±3.31sec/h, n = 16 rats) compared to naïve control rats treated with either vehicle (C+V), (0.60±0.37 sec/h, n = 16), or rapamycin (C+R), (1.45±0.58 sec/hr, n = 15). In contrast, rapamycin treatment in HS rats (HS+R) shows significant protection (1.48±0.42sec/h, n = 28, p<0.01). *: p<0.05, **: p<0.01, ***: p<0.001. Error bars indicate S.E.M.

Given these subacute protective effects of rapamycin, we further evaluated possible protective effects of mTORC1 inhibition on later life spontaneous seizures development. HS seizures were induced at P10, and rapamycin was administered as previously described. Video-EEG recordings were performed in young adult rats at P36–38. The fairly brief electrographic seizures (all groups: 6.4±0.71 sec; vehicle-treated HS group: 7.6±1.37 sec; Figure 7A) were accompanied by sudden behavioral arrest, staring episodes, head jerking, and facial automatisms. Consistent with our previous reports [15], we found that both the number of spontaneous seizure per hour and the cumulative time seizing per hour were significantly increased in vehicle-treated HS rats, relative to normoxic vehicle-treated littermates (p<0.01) (Figure 7B,C). Rats that had been previously treated with rapamycin around HS demonstrated significantly decreased later life spontaneous seizure frequency and reduced cumulative seizure time (p<0.01), relative to vehicle-treated HS rats. Importantly, treatment with rapamycin at P10 in normoxic rats did not cause any later life EEG changes compared to vehicle-treated controls, which at baseline have very low rates of EEG abnormalities (0.12±0.07 seizures/h, p>0.05) (Figure 7B,C), consistent with prior reports in normal Long-Evans rats [15], [45]. These data indicate that seizure-induced mTORC1 activation plays a critical role in causing later epileptogenesis after early life seizures, and that rapamycin treatment, associated with post-seizure suppression of mTORC1 activation, not only results in a decrease in subacute network hyperexcitability, but also can attenuate long term risk of epilepsy.

### Autistic-like Social Deficits Induced by Neonatal Hypoxic Seizures are Reversed by mTORC1 Inhibition

**Figure 8:**
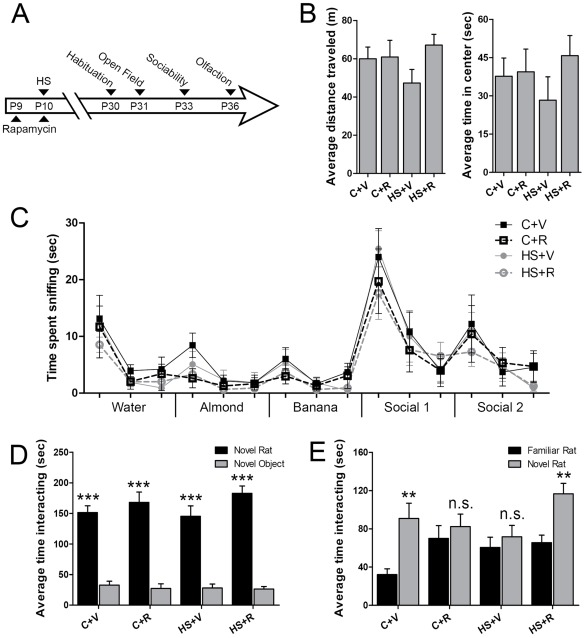
Rapamycin attenuates long term behavioral deficits after neonatal seizures. A. Schematic of behavioral testing protocol B. Distance traveled and time spent exploring the center zone during a 30-minute open field test does not vary significantly between the treatment groups (n = 16–29, p>0.05) (C+V: vehicle-treated sham control litter mates; C+R: rapamycin-treated sham control litter mates; HS+V: vehicle-treated HS rats; HS+R: rapamycin-treated HS rats). C. The olfactory habituation/dishabituation assay confirms no significant differences in the time spent sniffing non-social odors or social odors, with similar habituation and dishabituation, as the time spent sniffing the first swab of each new odor increases (two-way ANOVA for treatment [F(3,420) = 1.18, p = 0.32], odor [F(14,420) = 22.59, p<0.0001] and interaction [F(42,420) = 0.33, p = 1.00]). D. All groups spent significantly more time interacting with the novel rat than with a novel object in the three-chamber sociability test (two-way ANOVA for treatment [F(3,146) = 0.58, p = 0.63], stimulus [F(1,146) = 255.9, p<0.0001] and interaction [F(3,146) = 1.32, p* = *0.27]). E. In contrast, there were significant differences in the preference for social novelty among treatment groups (Bonferroni post-tests). C+V rats had a significant preference for social novelty, spending greater time interacting with the novel rat (90.88±15.93 vs. 32.13±6.09 sec, n = 16, p = 0.0013), while the HS+V rats lacked a preference for social novelty (76.97±14.11 vs. 50.73±9.76 sec, n = 16, p = 0.14). Importantly, rapamycin treatment reversed the deficits in social novelty in HS rats (HS+R), (113.24±10.67 vs. 70.74±8.41 sec, n = 29, p = 0.0017). Rapamycin-treated control rats (C+R) did not significantly shift their preference for the social novelty (82.46±12.99 vs. 69.96±13.52 sec, n = 16, p = 0.49) (two-way ANOVA for treatment [F(3,146) = 3.41, p = 0.02], stimulus [F(1,146) = 17.21, p<0.0001] and interaction [F(3,146) = 1.29, p* = *0.28]).

Given the link between epilepsy and autism, we aimed to identify whether altered social behavior could be detected following early life seizures, and secondly whether this could be modified by rapamycin treatment. Standardized behavioral assays were used to measure social behavior (Figure 8A) [46], [47], [48], [49]. First, open field and olfactory habituation/dishabituation tests were performed and confirmed no baseline differences between groups, ruling out any primary differences or alterations in motor and sensory function in the treated HS and control rats (Figure 8B,C). Next, social behavior assessed using a three-chamber social choice test showed a significant preference for sociability in all four groups, indicated by greater time spent with a social stimulus than a non-social object (p<0.0001, Figure 8D). In contrast, the preference for interacting with a novel rat over a familiar rat was significantly reduced in vehicle-treated HS animals (p = 0.14) compared with the significant preference for the novel rat exhibited by naïve controls (p<0.002, Figure 8E), indicating that early life seizures can impair later social behavior to an extent similar to other models of autism [47], [50]. Furthermore, rats that had been treated with rapamycin were protected against exhibiting later deficits, and showed significant levels of preference for social novelty (p<0.002), suggesting an important role of mTORC1 pathway activation in inducing altered social behavior following early life HS. Interestingly, the brief treatment of sham control rats at P10–11 with the same doses of rapamycin appeared to cause a loss of preference for the social novelty (p = 0.49), suggesting that even a 24 h disruption of mTORC1 activity during the critical period is important for normal social development.

## Discussion

Early life epilepsy is associated with increased risk of intellectual disability and autism [12] and epilepsy occurs at a significantly higher rate in autistic patients relative to the general population [7], [8]. The interaction between epilepsy and autism is likely to be multifactorial, but the present study supports a significant role for perturbations in mTORC1 signaling pathway in linking the pathogenesis of these two disorders, both of which have been attributed at least in part to synaptic dysfunction. Early life is characterized by enhanced synaptogenesis and synaptic plasticity, and here we show that there is a commensurate increase in activation of mTORC1-dependent downstream signaling in the immature brain. While mTORC1 has been shown to regulate synaptic function and plasticity *in vitro* [51], [52], we now demonstrate that seizures sequentially activate up- and downstream components of plasticity related mTORC1 signaling, including upstream activators PI3K/Akt, MAPK/ERK and BDNF and downstream effectors 4E-BP1, p70S6K and ribosomal protein S6. The mTORC1 pathway is therapeutically targetable, and a major finding of this study is that pharmacologic suppression of mTORC1 activity with acute rapamycin treatment disrupts subsequent development of epilepsy as well as autistic-like behavior, suggesting that the mTORC1 pathway may be a common, and reversible, mediator for the interaction between early life epilepsy and autism.

This is the first report to show that markers of mTORC1 activity are transiently increased at baseline during the first three postnatal weeks in rodents, consistent with heightened synaptic plasticity. The expression of phosphorylated p70S6K (Thr389), one of the best-characterized downstream targets of mTORC1 [17], was highest during the first postnatal weeks in both hippocampus and neocortex. The increased mTORC1 signaling appears to be occurring coincident with the developmental upregulation of known upstream activators of this pathway, including glutamate receptors, BDNF and Rheb [39], [53], [54], [55], which supports its critical involvement in synaptic and network development [56]. Interestingly, we found a developmental time lag between the increase in phospho-p70S6K and phospho-S6. To date, the timing of activation of these pathways has only been studied in cell cultures or acute slices, and our *in vivo* results are especially intriguing given the reported rapid kinetics of these phosphorylation events *in vitro* [41], [57]. The activity of mTORC1 is highly regulated and balanced: for example, activation of p70S6K blocks further activation of this pathway by inhibiting PI3K/Akt signaling [58]. During development, this negative feedback loop might be initially stronger then the ability of p70S6K to activate S6, at least to a level detectable by western blot in brain tissue. Interestingly, the delay in S6 activation was more pronounced in the neocortex, where phospho-p70S6K levels were highest. Another interesting finding was the apparent disconnect between the steady increase in total mTOR protein and the downregulation of mTORC1 signaling with increasing age, which would suggest that in the adult brain, the activity of mTORC2 predominates over mTORC1. This hypothesis is supported by the developmental regulation of phospho-mTOR (Ser2448) showing a progressive increase with maturation, at least in the hippocampus. Although the functional relevance of this phosphorylation site in neurons remains to be determined, it has been previously reported that phospho-mTOR (Ser2448) can be associated with both mTORC1 and mTORC2 complexes [37]. Future analyses, beyond the scope of this study, will need to examine the developmental regulation of specific mTORC2 targets, including phospho-Akt (Ser 473) [59].

**Figure 9:**
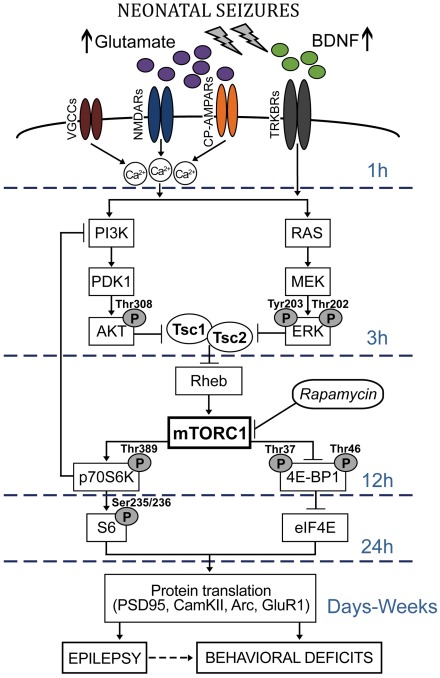
Potential mTORC1-dependent mechanisms involved in epileptogenesis and altered social behavior following neonatal seizures. Neonatal seizures induce intense synaptic activity followed by rapid increases in extracellular glutamate and BDNF levels (within 1 h post-HS; Figure 4A). Activation of glutamate receptors, including NMDA receptors (NMDARs) and Ca^2+^-permeable AMPA receptors (CP-AMPARs) [44], followed by opening of L-type voltage-gated Ca^2+^ channels (VGCCs), results in increased Ca^2+^ influx. Enhanced expression and function of BDNF activates the membrane-bound tyrosine kinase B receptors (TRKBRs) and, together with Ca^2+^ influx, leads to the recruitment of the PI3K/PDK1/Akt and the Ras/MEK/ERK signaling pathways (within 1–3 h post-HS; Figure 4B–C). Akt- and ERK-dependent inactivation of Tsc1/Tsc2 complex results in activation of Rheb GTP-ase, and subsequent induction of mTORC1 activity (within 12–24 h post-HS; Figure 3). In turn, the mTORC1 may inhibit 4EBP1 and enhance the activity of p70S6K at 12 h post-HS, leading to enhanced eIF4E and S6 signaling, and increased protein translation at 24 h post-HS. The negative feedback inhibition between p70S6K and PI3K may account for the time lag between p70S6K and S6 phosphorylation changes. Altered TORC1-dependent translation of synaptic plasticity proteins (such as PSD-95, CamKII, Arc, GluR1) [40], [91], among with other important cellular processes regulated by the mTORC1 kinase (autophagy, ribosomal biogenesis and cellular metabolism) [92], may lead to both short-term and long-term neuronal hyperexcitability (Figure 6B), enhancement of synaptic function (Figure 6C-F), epilepsy (Figure 7), and autism-like behaviors (Figure 8). Treatment with the mTORC1 inhibitor rapamycin immediately before and after neonatal seizures reverses both early and late neurological and behavioral consequences (Figures 6–8).

This study also reveals upregulation of multiple mTORC1 pathway components following neonatal seizure induction in both the hippocampus and neocortex. Specifically, we found that mTORC1 downstream signaling is transiently activated, likely through induction of the putative upstream activators PI3K/Akt, MAPK/ERK and BDNF pathways (Figure 9). This is consistent with synaptic plasticity models [40], [41], and status epilepticus in adult rats [34], [60]. We show a sequential transient increase in BDNF, phospho-Akt and phospho-ERK between 1 and 3 h after seizure induction, followed by upregulation of the mTORC1 downstream effectors phospho- 4E-BP1, phospho-p70S6K and phospho-S6 at 12–24 h post-seizure (Figure 9).

Seizures can rapidly elevate both glutamate and BDNF levels in adult and immature animal models of epilepsy [61], [62], [63], which is in agreement with our findings. Interestingly, in addition to the early increases of BDNF in hippocampus and neocortex, we found a later upregulation at 12 h in hippocampus, which is consistent with the fact that multiple waves of activity-induced BDNF-dependent protein synthesis are required for hippocampal memory consolidation, beyond the first few hours post-training [29], [64]. BDNF, in addition to its role in neuronal proliferation, differentiation and survival, represents a crucial regulator of synaptic function and plasticity, in particular, it can activate the PI3K/Akt and MAPK/ERK pathways promoting dendritic growth, dendritic branching, and spine formation [65], [66].

Phospho-Akt and phospho-ERK may be important in promoting epileptogenesis, beyond their function of activating mTORC1. Akt has an important role in cell proliferation and cell survival [67] and may have a neuroprotective role in this model. ERK signaling can enhance protein synthesis by directly phosphorylating S6 at Ser235/236 [68] as well as p70S6K at multiple sites [41]. In addition, ERK can modulate the transcription of multiple plasticity genes by activating the transcriptional regulator cAMP response element-binding CREB [69]. Interestingly, we found that phospho-ERK responses were more robust in the hippocampus, consistent with its critical role in hippocampal synaptic plasticity, learning and memory [70], [71], [72].

Notably, the activation of downstream mTORC1 signaling in our mild neonatal seizures model occurs somewhat later than the reported phospho-S6 upregulation following induction of status epilepticus in adult rats (1–6 h post) [32], [34]. In addition to more severe seizures, the adult models also cause neuronal death, whereas our neonatal model exhibits no cell death and shows ongoing subclinical excitability over the first 24 to 48 h after seizure onset [15], [16]. Furthermore, hypoxia was used to induce neonatal seizures in this study, and it is known that hypoxia itself can suppress mTORC1 signaling [73], [74], [75]. Although the duration of hypoxia used for seizure induction was only 15 min, which is much less then needed to induce a marked inhibition of phospho-p70S6K [75], we cannot exclude that this may have prolonged the time lag between the seizure-induced activation of mTORC1 upstream and downstream components. Nevertheless, the lag in activation may present a window of opportunity for intervention.

Enhancing the significance of seizure-induced activation of mTORC1 in neonatal seizures, we found that *in vivo* rapamycin treatment attenuated a number of epileptogenic consequences previously reported in this model [11], [15], [36]. Effective inhibition of mTORC1 signaling at P10 during HS protected against enhanced susceptibility to KA-induced seizures at P13, but also reversed the long term effects of HS in inducing later life spontaneous seizures. Importantly, these effects were not due to suppression of acute HS, as their severity and duration were unaffected by rapamycin treatment. This is consistent with previous studies in adult rats that show little or no immediate effects of rapamycin on acute status epilepticus *in vivo* [32], [33], and *in vitro* studies showing no direct effect of rapamycin on excitatory synaptic transmission or neuronal excitability [76], [77].

Rapamycin treatment not only blocked the increases in early mTORC1 activity markers and later seizure susceptibility, but also prevented seizure-induced changes in synaptic function. We have previously shown that epileptogenesis in this model is selectively mediated by enhanced activation and modification of AMPA receptor subtype of ionotropic glutamate receptors, as evidenced by HS-induced increases in AMPA receptor mEPSCs and sEPSCs [11], [16]. Importantly, treatment with AMPA receptor antagonists in the subacute period following HS prevents long term increased network excitability and impaired synaptic transmission [16], [36], [78]. Similarly, we found that rapamycin significantly attenuated the increased AMPA receptor mEPSCs and sEPSCs amplitude in HS rats, without affecting the basal synaptic transmission. Notably, components of the mTORC1 pathway co-localize with post-synaptic scaffolding protein post-synaptic density 95 (PSD95), facilitating local long-lasting changes in synaptic function [30]. mTORC1 regulates translation of immediate early genes, and of genes encoding ion channels, protein kinases or cytoskeletal proteins critical to spine and synapse function [29], [40], [79], [80]. As the mRNAs encoding glutamate receptor subunits are localized in dendrites and can undergo local translation in an activity-dependent manner [81], activation of mTORC1 could serve to increase local translation of AMPA receptor subunits themselves, or of related proteins involved in their trafficking and insertion [28]. This is consistent with our data showing the co-occurrence of increased phospho-S6 in dendrites of pyramidal neurons at 24 h post-HS and subacute increases in AMPA receptor-mediated sEPSC and mEPSCs amplitude.

This study provides the first evidence for development of autistic-like social behavior deficits following neonatal seizures in wild type animals, manifested as a lack of preference for social novelty. Similar social novelty deficit have been shown in several autism mouse models [50], [82], [83], [84], including genetic models that involve dysregulation of mTORC1 pathway components [25], [47]. Although currently there is no known casual relationship between epilepsy and autism [7], the high association of these two disorders suggests that they may share some anatomical and molecular mechanisms [7], [9], [44], [85]. The same *in vivo* rapamycin treatment that prevented subacute changes in seizure susceptibility and long term epilepsy also ameliorated these autistic-like behavioral deficits. Thus the present study implicates the mTORC1 pathway not only in epileptogenesis, but also in the behavioral consequences of early life seizures. These results raise the possibility that modifying epileptogenesis in the immature brain may also prevent other manifestations of network dysfunction involved in later life neurobehavioral co-morbidities. While these results suggest a disease modifying effect of rapamycin for both epilepsy and secondary autism, caution must be placed on potential clinical translation. While rapamycin showed a beneficial effect in post-seizure animals, there was a mild effect on later social preferences in control rats that had been treated at P10. Hence the protection here is predicated on pathological increases in mTORC1 activity by early life seizures, and in the absence of this pathological upregulation, suppression of the mTORC1 pathway may have actually caused subtle developmental abnormalities in the naïve controls [86]. Safety studies of mTORC1 inhibition must address these issues.

In conclusion, these results show a time-dependent activation of up and downstream components of mTORC1 pathway, beginning within an hour after seizure onset and lasting up to 24 h in the immature rat brain (Figure 9). Activation of the mTORC1 pathway, and subsequent increased AMPA receptor function, may have a critical role in epilepsy as well as autistic-like behavior as a consequence of early life seizures and may be one of probably multiple convergence points underlying an interaction between autism and neonatal seizures (Figure 9). These results suggest that this interaction is not simply limited to TSC patient population, and that mTORC1 inhibition may have more widespread application as a preventative, disease modifying therapeutic strategy following refractory early life seizures with impact on subsequent brain function.

## Materials and Methods

### Animals

Male Long-Evans rat pups (Charles River Laboratories, Wilmington, MA) were maintained in a 12 h light/dark cycle facility. All experiments were approved by the Animal Care and Use Committee at Children’s Hospital (Boston, MA), and were in accordance with the National Institutes of Health guidelines.

### Hypoxia-induced Seizures

Hypoxic seizures (HS) were induced in Long Evens rats at postnatal day (P) 10. Briefly, pups were exposed to graded global hypoxia for 15 min (7% O_2_ for 8 minutes, 5% O_2_ for 6 minutes, 4% O_2_ for 1 minute), as described previously [13]. Under hypoxic conditions, over 95% pups experience tonic-clonic seizures, automatisms followed by head and limb movements, and myoclonic jerks, while only a very small number of animals (<5%) that are exposed to hypoxia do not respond with seizures. Seizures develop gradually during the 15 min hypoxia, however rat pups continue to present short spontaneous seizures over 48 h following the initial hypoxic insult [15]. All experimental groups have experienced the same level of hypoxia for the same period of time. Littermate controls went through the same procedure, but exposed to normal air.

### Administration of Rapamycin

Rapamycin (LC Laboratories, Woburn, MA) was dissolved in 100% ethanol and diluted in 5% Tween 80, 5% polyethylene glycol 400 (Sigma) and 4% ethanol before use. To time rapamycin administration, we first established its pharmacokinetics in P9 rats, based on the effect of a single dose (3 mg/kg) to suppress the mTORC1 pathway marker phospho-S6 (Ser235/236). Western blots performed on cortical tissue to assess levels of phospho-S6 at 1 h, 6 h, 12 h, 24 h, and 48 h following a single injection of rapamycin revealed that maximal phospho-S6 suppression was obtained at 24 h following injection (1.7±0.4%, p<0.05), with partial reduction as early as 1 h post-injection (19.7±4.8%), and almost restored levels at 48 h. These data correlate with pharmacokinetic studies in solid tumors after systemic rapamycin administration, where trough levels of phospho-S6 were found at 24–48 h [87], [88]. These data suggest that pre-treatment is necessary in order to achieve optimal mTORC1 pathway inhibition at the time of insult and/or over the first 24 h of a seizure induction. This is also suggested by prior publications demonstrating the increased efficacy of rapamycin in a pre-treatment paradigm, as opposed to post-seizure treatment, for suppression of long term epileptogenesis [43], [89]. Therefore, control and HS rats were treated with rapamycin (3 mg/kg i.p.) or vehicle 24 h before and 1 h after exposure to hypoxia at P10.

### Kainic Acid (KA)-induced Seizures

At P13 (72 h post-HS), control and HS rat pups previously treated with either vehicle or rapamycin were administered KA i.p. (2 mg/kg, Cayman Chemical, Ann Arbor, MI). Behavioral seizures were videotaped for a 3 h period. Seizure severity (maximal seizure stage reached graded from 0 to 5) was evaluated blinded by two independent investigators, as described previously [11], [36]. Differences in seizure severity among groups were analyzed by one-way ANOVA followed *post hoc* Bonferroni multiple comparison procedures. Statistical significance was defined as *p*<0.05.

### Western Blot Analysis

For time course studies, male Long-Evans rats were euthanized at P3, P6, P10, P11, P16, P21 and adulthood (P50 and older, average weight 300–400 g) (n = 3–7/age group). HS and littermate control pups were euthanized at 1 h, 3 h, 12 h, 24 h, and 48 h after seizures (n = 4–21/group). Western blots were performed as previously described [11]. Briefly, hippocampal and cortical tissue was dissected, rapidly frozen in ethanol and stored at –80°C until used for whole-cell protein preparations. Tissue was homogenized in lysis buffer containing a Complete Mini Protease Inhibitor Cocktail Tablet (Roche, Germany) and the phosphatase inhibitors phenylmethanesulfonyl fluoride (1 mM), sodium-orthovanadate (1 mM) and okadaic acid (0.1 mM) to block proteases and phosphatases. Total protein concentrations were measured using a Bradford protein assay (Bio-Rad, Hercules, CA), and samples were diluted for equal amounts of protein in each lane. Whole-cell proteins were electrophoresed onto 4–20% Tris-HCl gels, 12% Bis-Tris gels or 3–8% Tris-Acetate gels and were subsequently transferred to polyvinylidene difluoride membranes (Bio-Rad). Immunoblots were incubated with primary antibodies at 4°C overnight. Except for BDNF (1∶100, Santa Cruz Biotechnology, Santa Cruz, CA), all antibodies were purchased from Cell Signaling (Beverly, MA): phospho-Akt (Thr308) (1∶500), Akt (1∶500), phospho-Erk1/2 (Thr202/Tyr204) (1∶500; 1∶1000), Erk1/2 (1∶500; 1∶1000), phospho-4E-BP1 (Thr37/46) (1∶100; 1∶500), 4E-BP1 (1∶100; 1∶500), phospho-p70S6K (Thr389) (1∶300; 1∶500), p70S6K (1∶500), phospho-S6 (Ser235/236) (1∶500; 1∶1000), S6 (1∶500; 1∶1000) and mTOR (1∶500). Membranes were then incubated with horseradish peroxidase -conjugated anti-rabbit IgG secondary antibodies (1∶5000, Pierce, Rockford, IL). Protein bands were visualized with enhanced chemiluminescence (Pierce) and measured with the Image Reader LAS-3000 system and Image Gauge v3.0 software (Fujifilm). To control for differences in protein loading, raw values were normalized to corresponding β-actin (Sigma-Aldrich Corp., St. Louis, MO) within an individual age group or time point. Normalized values were expressed as a percent or ratio of the mean expression level of controls (either adult or normoxic control tissue run on the same blot). Phospho-protein/total protein ratios were calculated in a similar fashion. One-way ANOVA followed by *post hoc* Bonferroni procedures were used for multiple comparisons across development. For the post-seizure time-course experiments two tailed t-tests were used, as each time point was statistically its own experiment. Statistical significance was defined as p<0.05.

### Immunocytochemistry

Control and HS pups were sacrificed at 24 h post-seizures. Brains were collected and processed following standard protocols [24], [39]. Briefly, 4% paraformaldehyde-fixed brains were sectioned at 50 µm on a Microm freezing microtome and stained with neuronal markers NeuN (1∶100, Millipore, Billerica, MA) and non-phosphorylated neurofilament (SMI 311, 1∶1000, Covance, Princeton, NJ), GABA-ergic marker GAD67 (1∶1000, Millipore), astrocytic marker GFAP (SMI 22, 1∶1000, Covance) and phospho-S6 (Ser235/236) (1∶500, Cell Signaling) antibodies. Fluorescent conjugated secondary antibodies (Alexa Fluor 568 goat anti-mouse IgG and Oregon Green 488 goat anti-rabbit IgG, Molecular Probes, Eugene, OR) were used at concentrations of 1∶1000. Additional sections were incubated with omission of one or both primary antibodies to exclude false-positive labeling. Slides were viewed with an epifluorescence microscope (Nikon Eclipse 80*i*). Images were captured with a Q Imaging digital camera and the NIS-Elements BR software 3.0 (Micro Video Instruments). Confocal multiple plane images, collected at 1 µm intervals, were captured using a Zeiss LSM 510 scanning laser microscope (Germany) to acquire z-stacks.

### Hippocampal Slice Preparation

Control and HS male Long-Evans rat pups previously treated with either vehicle or rapamycin were decapitated at 48 h post-seizure. Hippocampal slices were prepared and maintained as previously described [11]. The brains were rapidly removed, glued to the stage of a vibrating blade vibratome (LEICA VT1000S, Leica Microsystem Inc., Bannockburn, USA), and submerged in ice-cooled (0–4°C), oxygenated (95% O_2_ and 5% CO_2_) cutting solution containing (mM) 210 sucrose, 2.5 KCl, 1.02 NaH_2_PO4, 0.5 CaCl_2_, 10 MgSO_4_, 26.19 NaHCO_3_, and 10 D-glucose, pH 7.4. Coronal slices (300 µm thickness) of the middle third of the hippocampus were sectioned in cutting solution. Slices were then incubated in oxygenated artificial cerebrospinal fluid (ACSF) at 35°C for 30 min and subsequently maintained at room temperature for at least 1 h before electrophysiological recordings. The ACSF contained (in mM): NaCl, 124; KCl, 5; NaH_2_PO4, 1.25; MgSO_4_, 1.2; NaHCO_3_, 26; CaCl_2_, 2; and glucose, 10, pH 7.4. For recordings, the slices were transferred to a 2.5 ml recording chamber placed in an upright Nikon Eclipse E600FN microscope equipped with infrared and differential interference contrast imaging devices and with a 40x water immersion objective, and superfused (1.0–1.5 ml/min) with gassed ACSF at room temperature (22–24°C).

### Whole-cell Patch-clamp Recordings

Whole-cell voltage clamp recordings were obtained from CA1 pyramidal neurons in hippocampal brain slices by using an Axopatch 200A amplifier (Molecular Devices, Silicon Valley, CA) and were performed at room temperature (22–24°C). Patch electrodes were prepared from borosilicate glass capillaries with a Flamming/Brown micropipette puller (Model P-87, Sutter Instruments Co., Novato, CA, USA). The patch-pipettes had a resistance of 3–6 MΩ when filled with the internal solution that contained (in mM): 110 Cs-methanesulfonate, 10 TEA-Cl, 4 NaCl, 2 MgCl_2_, 10 EGTA, 10 HEPES, 4 ATP-Mg, and 0.3 GTP, with 5 QX-314 (*N*-(2,6-dimethylphenylcarbamoylmethyl)-triethylammonium chloride) and creatine phosphokinase (17 unit/ml). The pH of the pipette solution was adjusted to 7.2–7.3 with CsOH and the osmolarity was 280–290 mOsm/kg. AMPA-receptor-mediated spontaneous EPSCs (sEPSCs) were pharmacologically isolated by adding picrotoxin (60 µM) and DL-AP-5 (100 µM) to perfusion solution to block GABA and NMDA receptors, respectively. To record AMPA receptor-mediated miniature EPSCs (mEPSCs), TTX (1 µM) was added to the ACSF. Series resistance was compensated 70–80% and monitored online. Only recordings in which the series resistance was <25 MΩ and less than 15% change during the whole recording period were included in the data analysis. Signals were filtered at 2 KHz, digitized at 20 kHz by a Digidata 1320A interface, acquired by the pClamp 9.2 software, and analyzed with the Clampfit 9.2 program (Molecular Devices). sEPSC and mEPSC events at the holding potential of -60 mV were detected automatically with a threshold of 5–6 pA, depending on the noise level. All events were confirmed visually on the basis of the rise and decay times. The cumulative distributions of the sEPSCs were constructed from at least 10 min of recording from each cell, using a bin width of 1 pA for amplitude. Statistical significant differences were established at p<0.05 using the Kolmogorov-Smirnov test, or ANOVA followed by *post hoc* Bonferroni multiple comparison procedures.

### Video-EEG Recordings with Implanted Subcutaneous Electrodes

Video-EEG recordings 3–4 h in duration were acquired from P36–38 rats previously assayed in the behavioral testing. A total of over 280 rat-hours of video-EEG data were analyzed, which provided a good aggregate profile of spontaneous seizure activity, despite the short recording time in each individual animal (vehicle-treated control: 52 h, n = 16; rapamycin-treated control: 60 h, n = 15; vehicle-treated HS: 57 h, n = 16; rapamycin-treated HS: 115 h, n = 28). Teflon-coated silver/silver chloride subdermal wire electrodes were implanted subcutaneously in rats lightly anesthetized with 2–4% isoflurane. The rats were connected to a low torque commutator (Dragonfly Inc, Ridgeley, VA, USA) and connector assembly (John Ives, Manitock, ON, Canada) and were able to move freely around the enclosure [15]. Seizures were defined by the appearance of sustained polyspike activity, significantly different than background rhythm, longer than 3 sec, and associated with a behavioral correlate on video (sudden behavioral arrest, staring episodes, head-jerking, and facial automatisms). Average seizure frequency was calculated per hour of video-EEG recording, and seizure duration was measured as the time from first spike to last spike. Statistical significant differences were established at p<0.05 using non-parametric Kruskal-Wallis and Dunn’s multiple comparison tests.

### Behavioral Testing

Control and HS rat pups treated with rapamycin or vehicle were tested as adults in the following three assays: 1) open field locomotion, 2) three-chamber social choice test and 3) olfactory habituation/dishabituation. Behavioral experiments were conducted in a standard behavioral testing room during the light phase (0700–1900 h) of a 12 h light-dark cycle [46]. All investigators were blind to subject treatment groups. Statistically significant differences were established at p<0.05 using ANOVA followed by *post hoc* Bonferroni tests or non-parametric Kruskal-Wallis tests.

*Open field locomotion:* The day before testing a P29–30 rat was habituated for three hours in a 100x100x35 cm opaque Plexiglas chamber (Stoelting, Co., Wood Dale, IL, USA). The following day, each P30–31 rat was placed in the center of the open field, illuminated at 120–129 lux, for 30 minutes and was tracked using ANY-maze Video Tracking System (Stoelting, Co.). Data on distance traveled and time spent in the center 80x80 cm area was automatically calculated by the software. The chamber was cleaned with Clidox®-S, (PRL Pharmacal Research Laboratories, Inc., Naugatuck, CT, 1∶18∶1) and 70% ethanol and was allowed to dry completely between test sessions [90].

*Three-chamber social choice test:* On the day of testing, an experimental P33–34 rat was individually housed in the behavior room 30 minutes prior to testing. The rat was placed in the center chamber of a 100x100x35 cm clear Plexiglas three-chamber maze (Stoelting, Co.), illuminated at 35–55 lux, and was allowed to habituate for ten minutes. The doors to both side chambers were then opened for the rat to explore all three chambers for ten minutes. The sociability test commenced with a stranger rat social stimulus (novel rat) confined within a wire cage in one side chamber and a nonsocial stimulus of an identical empty wire cage in the opposite side chamber. The experimental rat was then allowed to explore all three chambers for ten minutes. The social novelty test began immediately afterwards and the experimental rat was allowed to approach and sniff the previously investigated social stimulus (familiar rat) and a novel social stimulus of a second stranger rat (novel rat) in the side chambers for ten minutes. Transitions between chambers and time spent in each chamber were automatically recorded using the ANY-maze System. Time spent sniffing the empty cage, novel rat, and familiar rat were each recorded by an investigator in ANY-maze based on the duration of a keypress on a computer keyboard. The stranger rats were age- and gender-matched to the experimental rats, and were habituated to the wire cages for at least an hour on the day prior to testing [46], [90]. The three-chamber maze and wire cages were cleaned with Clidox®-S (1∶18∶1) and 70% ethanol and were allowed to dry completely between test subjects.

*Olfactory habituation/dishabituation:* P36–38 rats were acclimated to testing conditions in a new, empty cage (45x24x20 cm) with clean bedding. A sterile cotton swab (15 cm, Puritan Medical Products Company LLC, Guilford, MN, USA) was dipped in water, inserted though the lid of the cage and stabilized with a binder clip so that the moistened cotton tip was positioned 9 cm above the floor of the cage. The rat was observed for two minutes and the amount of time spent sniffing the cotton swab was quantified with a stopwatch. Sniffing was defined as the rat positioning its nose within 1 cm of the cotton tip. This was repeated for two further two-minute trials with fresh water dipped swabs. Consistent with this method, each rat was exposed in triplicate to two additional nonsocial and two social odors on cotton swabs in the following order: 1) water, 2) almond extract, 3) imitation banana extract, 4) social odor #1 (soiled rat cage that contained unfamiliar male rats), 5) social odor #2 (soiled rat cage that contained different unfamiliar male rats). The almond and banana extracts (McCormick & Co., Inc. Hunt Valley, MD, USA; 1∶100 in tap water) were diluted freshly the morning of testing. The social odors were prepared by wiping the cotton swab along the perimeter and down the center of a rat cage that contained soiled bedding and unfamiliar male rats [90]. Video of each experiment was recorded and reviewed by two independent observers.

## Acknowledgments

The authors would like to thank Ms. Kathia Cordero for technical assistance. We would like to thank Dr. Jacquelyn Crawley for her advice regarding socialization testing in rats.
